# Neoadjuvant treatment *versus* upfront surgery in borderline resectable and resectable pancreatic ductal adenocarcinoma: meta-analysis

**DOI:** 10.1093/bjsopen/zrae172

**Published:** 2025-03-24

**Authors:** Luke D Dickerson, Jayden Gittens, Chris Brunning, Richard Jackson, Michael C Schmid, Ainhoa Mielgo, Daniel Palmer, Christopher M Halloran, Paula Ghaneh

**Affiliations:** Institute of Systems, Molecular and Integrative Biology, University of Liverpool, Liverpool, UK; Pancreatobiliary Surgery Unit, Liverpool University Foundation Trust, Liverpool, UK; Institute of Systems, Molecular and Integrative Biology, University of Liverpool, Liverpool, UK; Institute of Systems, Molecular and Integrative Biology, University of Liverpool, Liverpool, UK; Institute of Systems, Molecular and Integrative Biology, University of Liverpool, Liverpool, UK; Institute of Systems, Molecular and Integrative Biology, University of Liverpool, Liverpool, UK; Institute of Systems, Molecular and Integrative Biology, University of Liverpool, Liverpool, UK; Institute of Systems, Molecular and Integrative Biology, University of Liverpool, Liverpool, UK; Department of Medical Oncology, Clatterbridge Cancer Centre, Liverpool, UK; Institute of Systems, Molecular and Integrative Biology, University of Liverpool, Liverpool, UK; Pancreatobiliary Surgery Unit, Liverpool University Foundation Trust, Liverpool, UK; Institute of Systems, Molecular and Integrative Biology, University of Liverpool, Liverpool, UK; Pancreatobiliary Surgery Unit, Liverpool University Foundation Trust, Liverpool, UK

## Abstract

**Background:**

Pancreatic cancer prognosis remains poor despite advances in adjuvant treatment. Neoadjuvant treatment may improve survival and disease-free survival. This meta-analysis evaluates the outcomes for patients with borderline-resectable (borderline-resectable pancreatic cancer) or resectable disease (resectable pancreatic cancer) in randomized trials of neoadjuvant therapy *versus* upfront surgery.

**Methods:**

The review was performed according to PRISMA guidance. Articles were included from the start of the database until 1 May 2024. The primary outcome was overall survival. Secondary outcomes were progression-free survival, resection rate, R0 rate, N0 rate, vascular resection rate, surgical complications, significant adverse events and rates of adjuvant therapy. Data was collected from study manuscripts or through individual patient-level data extraction. Meta-analysis was performed using a random-effects model with subgroup comparisons for resectability status (resectable pancreatic cancer *versus* borderline-resectable pancreatic cancer) and treatment modality (chemotherapy *versus* chemoradiotherapy).

**Results:**

Nine trials were included representing 1194 patients. Four trials recruited borderline-resectable pancreatic cancer, four resectable pancreatic cancer and one both. Four trials reported chemotherapy, four chemoradiotherapy and one both treatments. Neoadjuvant treatment improved overall survival (HR 0.73, 95% c.i. 0.55 to 0.98; *P* = 0.001) and progression-free survival (HR 0.80, 95% c.i. 0.65 to 0.99; *P* = 0.041). Subgroup analysis demonstrated neoadjuvant treatment improved overall survival for borderline-resectable pancreatic cancer (HR 0.60, 95% c.i. 0.38 to 0.96) but not resectable pancreatic cancer (HR 0.90, 95% c.i. 0.63 to 1.28). The overall resection rate was lower in neoadjuvant treatment (72.6% *versus* 80.6%, RR 0.94, 95% c.i. 0.89 to 0.99; *P* = 0.020). R0 rate (43.8% *versus* 23.0%, RR 1.35, 95% c.i. 1.16 to 1.57; *P* = 0.002) and N0 rate (30.9% *versus* 15.0%, RR 2.03, 95% c.i. 1.50 to 2.74; *P* = 0.001) was improved in neoadjuvant treatment. Significant adverse events occurred more frequently in neoadjuvant treatment (56.1% *versus* 27.0%, RR 1.92, 95% c.i. 1.28 to 1.89; *P* = 0.007).

**Conclusion:**

Neoadjuvant treatment significantly improves survival in borderline-resectable pancreatic cancer but not resectable pancreatic cancer. It should be regarded as standard of care for these patients. Further work is needed to identify the optimum neoadjuvant regimen and a possible role in the treatment of resectable pancreatic cancer.

## Teaser

This comprehensive systematic review and meta-analysis of nine trials and 1194 patients comparing neoadjuvant treatments to upfront surgery demonstrates improved overall survival in neoadjuvant treatment arms. This benefit is pronounced in borderline-resectable patients but there is no difference between chemotherapy and chemoradiotherapy. R0 and N0 rates are improved whilst surgical complications and significant adverse effects are comparable.

## Introduction

Pancreatic cancer (PDAC) remains a surgical and oncological challenge. Despite progress in treatment options it has a poor prognosis relative to other solid organ tumours with a 13% 2-year survival rate in the UK^1,2^. Management options are dictated by the patient’s physiological and anatomical suitability for surgery, which considers the presence of metastatic disease and extent of vascular resection required^3^. Standard of care for technically resectable disease is surgical resection and 6 months of adjuvant chemotherapy. This has been demonstrated to improve 5-year overall survival (OS) in the ESPAC-4 cohort to 28.8% with adjuvant gemcitabine and capecitabine, and improved 5-year survival in the PRODIGE24 cohort to 43.2% with folinic acid, flurouracil, irinotecan and oxaliplatin (mFOLFIRINOX)^4–6^.

Tumour contact with vasculature surrounding the pancreas makes surgical management technically more difficult and confers poorer prognosis. This has led to the subdivision of tumours into resectable (R-PDAC), borderline-resectable (BR-PDAC) and locally advanced (LA-PDAC), though definitions vary (*Table S1*)^7–11^. The inclusion of biological markers such as carbohydrate antigen 19-9 levels further complicates the classification of BR-PDAC^12^. Advances in surgical techniques and oncological management have led to considerable debate over optimal treatment, particularly in BR-PDAC. Although studies have demonstrated that a proportion of these patients are technically resectable, disappointingly, around 80% of patients will quickly develop local recurrence or distant metastasis following surgery^13^.

Neoadjuvant treatment is utilized in other solid tumours to downstage the extent of local disease and to treat clinically undetectable micrometastases^14–16^. Another theoretical benefit is the greater likelihood for delivery and completion of systemic treatment in the neoadjuvant setting than the adjuvant, although trial evidence at present doesn’t appear to entirely support this^17^. This is particularly pertinent in pancreatic cancer, given that national adjuvant treatment rates are only 62%^18^. Potential downsides of neoadjuvant regimens are disease progression during the interval in which neoadjuvant systemic treatment is delivered and adverse effects from preoperative treatment.

This study comprehensively reviewed clinical trials of neoadjuvant treatments against upfront surgery and ascertained the benefit of different treatment types (chemotherapy *versus* chemoradiotherapy) and in different population groups (R-PDAC *versus* BR-PDAC).

## Methods

This systematic review and meta-analysis was performed in accordance with the Preferred Reporting Items for Systematic Review and Meta-Analysis statement (PRISMA) guidance^19^.

### Search strategy

Scientific publication databases were searched (PubMed, Scopus and Cochrane Central Register of Controlled Trials) for trials comparing neoadjuvant treatment options *versus* surgery in pancreatic cancer. Trials of BR-PDAC and R-PDAC cancers were included. Articles were considered from database inception until 1 May 2024. Full search terms and inclusion/exclusion criteria are included in the *Supplementary material* (*Table S2*).

Inclusion criteria were randomized clinical trial (RCT), immediate surgery as one of the comparator groups, with extractable survival outcomes and articles written in English. Trials were excluded if they solely addressed metastatic disease or did not have a surgical comparator arm. Other research types including cohort studies, case series and systematic reviews/meta-analysis were excluded.

### Data collection and outcomes

Two authors (L.D. and C.B.) independently screened articles and extracted data from all included studies along with linked published analysis and protocols which was validated by a third reviewer (P.G.). OS was the primary outcome, and secondary outcomes were the trial reported marker of disease progression (progression-free survival (PFS)), resection rate, R0 resection rate, N0 rate, vascular resection rate, postoperative complication rate, serious adverse events (SAEs) and rates of starting adjuvant therapy^20,21^. Time-to-event outcomes were directly taken from articles where comparisons were made. In cases where OS and PFS comparisons were not explicitly reported, indirect methods for extraction of individual patient data (IPD) from Kaplan–Meier curves were utilized to create hazard ratios^22,23^. Complication rates and SAEs were reported per patient and included when Clavien–Dindo grade 3 or above and SAEs grade 3 or above respectively. Studies that did not report secondary outcomes in an analysable form had these outcomes excluded from pooled analysis. For subgroup analysis by resectability all trials had their resectability criteria reclassified as defined by the National Cancer Care Network Clinical Practice Guidelines (2017)^7^. Trials that included patients of both R-PDAC and BR-PDAC without presenting their results separately were classified as BR-PDAC.

Risk of bias was calculated using the Cochrane Risk of Bias 2 (RoB2) tool with low overall reported risk of bias in all domains^24^. Publication bias was assessed using funnel plots.

### Statistical analysis

All statistical analyses were performed in R v. 4.3.2 and a two-sided significance level of a *P* value less than 0.05 was used throughout for any explorative comparison. Meta-analysis was performed in R using *meta* (version 7.0-0) and *metafor* (version 4.6-0) packages using the random-effects model^25–27^. Subgroups were denoted by resection criteria (as per National Comprehensive Cancer Network (NCCN) criteria) and by neoadjuvant treatment modality (chemotherapy or chemoradiotherapy). Heterogeneity was assessed through *I*^2^, Tau^2^ and Cochrane's Q tests, with subsequent testing using influence analysis (Baujat and GOSH (Graphical Display of Study Heterogeneity) plots)^28,29^. Results are reported as hazard ratios (HR) for OS and PFS, and risk ratios (RR) for secondary outcomes with 95% confidence intervals. All outcomes were calculated using intention-to-treat (ITT) bar surgical complications (for which the denominator is attempted resection). For secondary outcomes, the overall numbers and percentages were calculated by treatment arms owing to completeness of the data for these subgroup analyses. Trials missing data on secondary outcomes were excluded from analysis of that secondary outcome.

## Results

### Study selection

A total of 1116 records were screened after the removal of duplicates. Seventy-eight articles were retrieved for full-text review, and 12 were included in the meta-analysis (seven trial paper reports^30–37^, two abstracts^38^, three articles reporting supplementary outcomes of interest^39–41^, *Fig. S1*).

### Study characteristics

Studies included were nine trials with 1194 patients randomized to upfront surgery (*n* = 592, 49.6%) or neoadjuvant treatment (*n* = 602, 50.4%). Of the neoadjuvant treatments, 389 (64.6%) were randomized to chemotherapy and 213 (35.4%) were randomized to chemoradiotherapy. Neoadjuvant and adjuvant regimens varied between trials and are described in *Table 1*.

**Table 1:** Included trial summary characteristics and details of treatment regimens

Trial name	Number of patients	Resectability	Treatment	Treatment details	
				Neoadjuvant	Adjuvant
Casedei^31^	38	R-PDAC	CRT	Gemcitabine (1 g/m^2^ on days 1 and 8 every 21 days for (2 cycles) then 50 mg/m^2^ twice weekly for 6 weeks alongside radiotherapy), conventional radiotherapy with 45 Gy and a boost of 9 Gy on the pancreatic lesion	Gemcitabine (1 g/m^2^ per week), 3-week treatment, 1 rest (6 cycles)
Golcher^30^	73	BR-PDAC	CTR	300 mg/m^2^ gemcitabine and 20 mg/m^2^ cisplatin (with radiotherapy days 1, 8, 22 and 28), total radiotherapy dose 1.8 Gy to 55.8 Gy	Gemcitabine (1 g/m^2^ per week), 3-week treatment, 1 rest (6 cycles)
Jang^32^	58	BR-PDAC	CTR	Gemcitabine 400 mg/m^2^ & 45 Gy in 25f & 9 Gy in 5f (5x/week for 6 weeks)	Gemcitabine (1 g/m^2^/week) 3-week treatment, 1 rest (4 cycles)
PACT-15^33^	88	R-PDAC	CTx	PEXG (IV cisplatin 30 mg/m^2^, epirubicin 30 mg/m^2^ and gemcitabine 800 mg/m^2^ on days 1 & 15 every 4 weeks with PO apecitabine 1250 mg/m^2^ OD 1–28 days) (3 cycles)	GEM adjuvant arm: gemcitabine (1 g/m^2^ per week), 3-week treatment, 1 rest (6 cycles)
					PEXG adjuvant arm: PEXG (6 cycles)
					Postneoadjuvant arm: PEXG (3 cycles)
Prep-02/JSAP-05^38^	364	R-PDAC	CTx	Gemcitabine (1 g/m^2^ on days 7 and 14) & S1 (40 mg/m^2^ daily 1–14 days) every 3 weeks (2 cycles)	S1 (40 mg/m^2^ daily on days 1–28 of 6-week cycle) if recovered by 10 weeks after surgery (4 cycles)
PREOPANC-01^34^	248	R-PDAC	CRT	Gemcitabine 1 g/m^2^ on days 1, 8, 15 followed by gemcitabine 1 g/m^2^ on days 1, 8 and 15 and 15f of 2.4 Gy over 3 weeks	Gemcitabine 1 g/m^2^ + nab-paclitaxel 125 mg/m^2^ weekly for 3 of 4 weeks (6 cycles)
		BR-PDAC			
ESPAC-5^35^	90	BR-PDAC	CTx*	Oxaliplatin 85 mg/m^2^, irinotecan 180 mg/m^2^ and folinic acid (at local practice) and fluorouracil 400 mg bolus day 1 and day 15 followed up by 2.4 mg/m^2^ infusion over 46 h every 2 weeks (4 cycles)	Predominantly gemcitabine based but given according to local guidelines Gemcitabine 12 (28%) GEMCAP 29 (67%) mFOLFIRINOX 2 (5%)
			CTx†	Gemcitabine 1 g/m^2^ weekly and capecitabine 830 mg/m^2^ BD for 21 days of 28 day cycle (2 cycles)	
			CRT	Capecitabine 830 mg/m^2^ BD for radiotherapy duration (5.5 weeks) 50.4 Gy over 28 fractions (1.8 Gy/fraction)	
NEONAX^36^	127	R-PDAC	CTx	Gemcitabine 1 g/m^2^ + nab-paclitaxel 125 mg/m^2^ weekly for 3 of 4 weeks (2 cycles)	Gemcitabine 1 g/m^2^ + nab-paclitaxel 125 mg/m^2^ weekly for 3 of 4 weeks within 12 weeks of surgery (6 cycles)
NORPAC-01^37^	140	R-PDAC	CTx	Oxaliplatin 85 mg/m^2^, irinotecan 150 mg/m^2^, leucovorin 400 mg/m^2^ and fluorouracil (2.4 g/m^2^ over 46 h), administered every second week on day 1 of each 14-day cycle (4 cycles)	mFOLFIRINOX (8 cycles if postneoadjuvant, 12 cycles if upfront surgery)

R-PDAC, resectable pancreatic cancer; BR-PDAC, borderline resectable pancreatic cancer; f, fractions; IV, intravenous; PO, per oral; OD, once daily; BD, twice daily; CTR, chemotherapy; CRT, chemoradiotherapy; CTx, chemotherapy; PEXG, cisplatin, epirubicin, gemcitabine and capecitabine; GEM, gemcitabine; GEMCAP, gemcitabine and capecitabine; mFOLFIRINOX, folinic acid, flurouracil, irinotecan and oxaliplatin. *mFOLFIRINOX. †GEMCAP.

Four trials included only patients with R-PDAC, four trials included patients with BR-PDAC, and one trial was comprised of both R-PDAC and BR-PDAC patients. A total of 517 patients had R-PDAC and 677 patients had BR-PDAC. Reclassification of two trials from R-PDAC to BR-PDAC was performed as they included patients with NCCN-defined BR-PDAC (Golcher and Unno)^7,30,38^.

In trials of neoadjuvant chemotherapy, gemcitabine was given in combination with nab-paclitaxel (NEONAX^36^), S1 (Prep-02/JSAP-05^38^), capecitabine (ESPAC-5^35^) or with cisplatin, epirubicin, gemcitabine and capecitabine (PEXG), (PACT 15^33^). Modified FOLFIRINOX (ESPAC-5^35^) and FOLFIRINOX (NORPACT-1^42^) were also utilized.

In trials of chemoradiotherapy, induction chemotherapy was with gemcitabine monotherapy (PREOPANC^34^, Casadei^31^ and Jang^32^), in combination with cisplatin (Golcher^30^), or as capecitabine monotherapy (ESPAC-5^35^). Conventional radiotherapy was used in all, with total radiation dose ranging between 36 and 55.8 Gy.

Adjuvant chemotherapy was given to all patients with macroscopically resected and histopathology confirmed PDAC, though treatment regimens varied (*Table 1*).

### Follow-up duration

Follow-up duration ranged between 12 months and 5 years with patients routinely being followed up at 3–6 monthly intervals for progression and survival. Specifics of trial follow-up durations and intervals can be seen in *Table 2*. Four trials were concluded early due to poor accrual rate in two^30,31^, outdated chemotherapy in one^33^ and superiority at interim analysis in one trial^32^.

**Table 2:** Table to demonstrate median survival outcomes, follow-up details and start point for time-to-event outcomes for included trials

Study name	Resectability	Arm	mOS (months)	mPFS (months)	Follow-up duration	OS Start point	PFS start point
Casedei^31^	R-PDAC	US	19.5	NR	7-year trial interval, no patients lost to f/u	Randomization	Treatment start date
		CRT	22.4	NR			
Golcher^30^	BR-PDAC	US	14.4	8.7	36 months (3 monthly for 2 years, 6 monthly 2+ years)	Randomization	
		CRT	17.4	8.4			
Jang^32^	BR-PDAC	US	12	NR	48 months (3 monthly)	Start of treatment (CRT or surgery depending on arm)	
		CRT	21	NR			
PACT-15^33^	R-PDAC	US*	20.4	4.7	Median f/u 54 months (i.q.r. 47.8–69.4)	Randomization	
		US†	26.4	12.4			
		CTx	38.2	16.9			
Prep-02/JSAP-05^38^	BR-PDAC	US	26.6	NR	5 years, (3 monthly for 2 years, 6 monthly for 2+ years)	Not specified	
		CTx	36.7	NR			
PREOPANC-01^34^	Both	US	14.3	7.7	Median 59 months (min. 35 months)—6 monthly for 2 years, then annually	Randomization	
		CRT	15.7	8.1			
	R-PDAC	US	15.6	9.3			
		CRT	14.6	9.2			
	BR-PDAC	US	13.2	6.2			
		CRT	17.6	6.3			
ESPAC-5^35^	BR-PDAC	US	10.7	8.9	12 months (3 monthly)	Randomization	Surgery
		CTx‡	NE	NE			
		CTx§	NE	NE			
		CRT	NE	NE			
NEONAX^36^	R-PDAC	US	16.7	5.9	3 years (3 monthly)	Randomization	
		CTx	25.5	11.5			
NORPACT-1^37^	R-PDAC	US	38.5	16.2	Median 22.7 months (i.q.r. 14.5–33.7)	Randomization	
		CTx	25.1	11.9			

R-PDAC, resectable pancreatic cancer; BR-PDAC, borderline resectable pancreatic cancer; mOS, median overall survival; PFS, progression free survival; NR, not recorded; NE, not evaluable; US, upfront surgery; CTx, chemotherapy; CRT, chemoradiotherapy; PEXG, cisplatin, epirubicin, gemcitabine and capecitabine; GEMCAP, gemcitabine and capecitabine; mFOLFIRINOX, folinic acid, flurouracil, irinotecan and oxaliplatin. *Adjuvant gemcitabine. †Adjuvant PEXG. ‡GEMCAP arm. §mFOLFIRINOX arm.

### Overall survival

OS rates were extracted from all trials. In eight trials, this was calculated from the point of randomization, and in Jang *et al*., it was calculated from start of first treatment^32^. Overall, neoadjuvant treatment improved survival (HR 0.73, 95% c.i. 0.55 to 0.98; *P* = 0.01). Median overall survival (mOS) was available for eight trials (11 comparisons). Survival varied between 12.0 and 38.5 months, with nine trials demonstrating neoadjuvant treatment had longer mOS (*Table 2*).

### Resectability subgroups

**Fig. 1:**
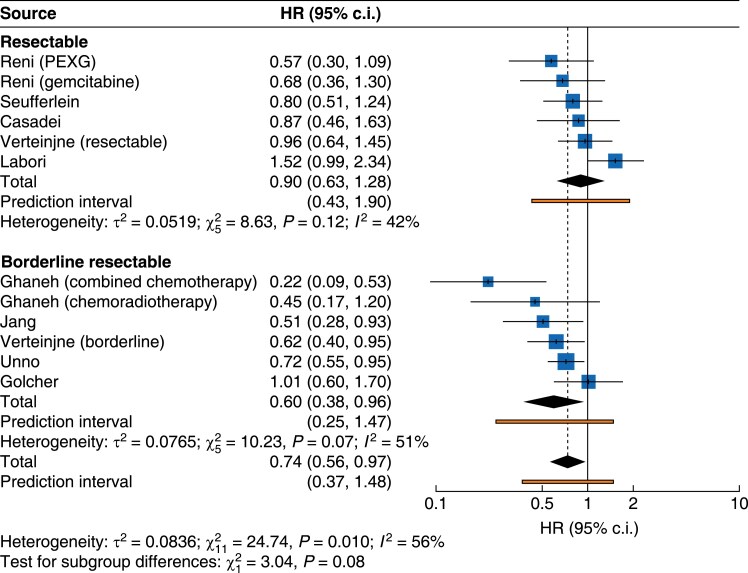
Forest plot demonstrating pooled analysis of overall survival with subgroup analysis according to resectability criteria (BR-PDAC *versus* R-PDAC) as per NCCN criteria BR-PDAC, borderline resectable pancreatic cancer; R-PDAC, resectable pancreatic cancer; NCCN, National Comprehensive Cancer Network; PEXG, cisplatin, epirubicin, gemcitabine and capecitabine.

Neoadjuvant treatment improved survival in patients classified as BR-PDAC (HR 0.60, 95% c.i. 0.38 to 0.96; *P* = 0.07). There was no statistically significant difference between subgroups for those with R-PDAC (HR 0.90, 95% c.i. 0.63 to 1.28; *P* = 0.12) (*Fig. 1* and *Table 3*).

**Table 3:** Summary statistics of pooled overall and progression-free survival for included trials

Pooled characteristics	Random-effects model			
	HR	95% c.i.	*I* ^2^	*P*
**Overall survival**				
Overall	0.73	0.55,0.98	55.8%	0.010
CRT	0.76	0.56,1.04	3.3%	0.762
CTx	0.70	0.38,1.29	72.9%	
BR-PDAC	0.60	0.38,0.96	51.1%	0.081
R-PDAC	0.90	0.63,1.28	42.0%	
Overall survival—outliers removed*				
Overall	0.74	0.64,0.85	0.0%	0.001
CRT	0.76	0.56,1.04	3.3%	0.611
CTx	0.72	0.61,0.84	0.0%	
BR-PDAC	0.69	0.52,0.92	2.2%	0.264
R-PDAC	0.80	0.63,1.02	0.0%	
**Progression-free survival**				
Overall	0.80	0.65,0.99	34.0%	0.041
CRT	0.87	0.67,1.14	0.0%	0.219
CTx	0.71	0.48,1.04	49.2%	
BR-PDAC	0.75	0.43,1.31	40.9%	0.799
R-PDAC	0.80	0.52,1.23	51.0%	
**Progression-free survival—outliers removed**†				
Overall	0.76	0.64,0.92	8.6%	0.009
CRT	0.87	0.67,1.14	0.0%	0.002
CTx	0.62	0.51,0.76	0.0%	
BR-PDAC	0.75	0.43,1.31	40.9%	0.860
R-PDAC	0.73	0.51,1.03	0.0%	

R-PDAC, resectable pancreatic cancer; BR-PDAC, borderline resectable pancreatic cancer; CRT, chemoradiotherapy; CTx, chemotherapy. *NORPACT-1 and ESPAC5 (combined chemotherapy) excluded. †NORPACT-1 excluded.

Post-hoc influence analysis demonstrated NORPACT-1 and the combined chemotherapy analysis of ESPAC-5 to contribute to influence and heterogeneity (*Fig. S2 a–d*). Exclusion of these from the analysis improved heterogeneity but did not affect the pooled results in the BR-PDAC (HR 0.69, 95% c.i. 0.52 to 0.92) or the resectable (HR 0.80, 95% c.i. 0.63 to 1.02) subgroups.

### Treatment subgroups

**Fig. 2:**
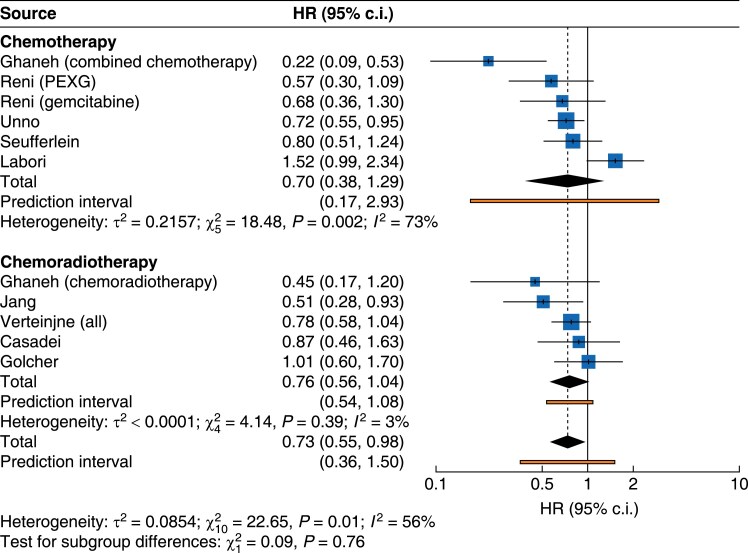
Forest plot demonstrating pooled analysis of overall survival with subgroup analysis according to treatment regimen (chemotherapy *versus* chemoradiotherapy) PEXG, cisplatin, epirubicin, gemcitabine and capecitabine.

There was no statistically significant improvement in OS in the subgroup analysis of chemotherapy (HR 0.70, 95% c.i. 0.38 to 1.29) and chemoradiotherapy (HR 0.76, 95% c.i. 0.56 to 1.04) (*Fig. 2* and *Table 3*).

Post-hoc analysis demonstrated the same trials (NORPACT-1 and combined chemotherapy of ESPAC-5) influenced the results. Exclusion of these trials demonstrated that neoadjuvant treatment improved survival for the cohort overall (HR, 0.74, 95% c.i. 0.63 to 0.85; *P* = 0.001) and for the neoadjuvant chemotherapy subgroup analysis (HR 0.71, 95% c.i. 0.61 to 0.84).

### Secondary outcome measures

**Fig. 3:**
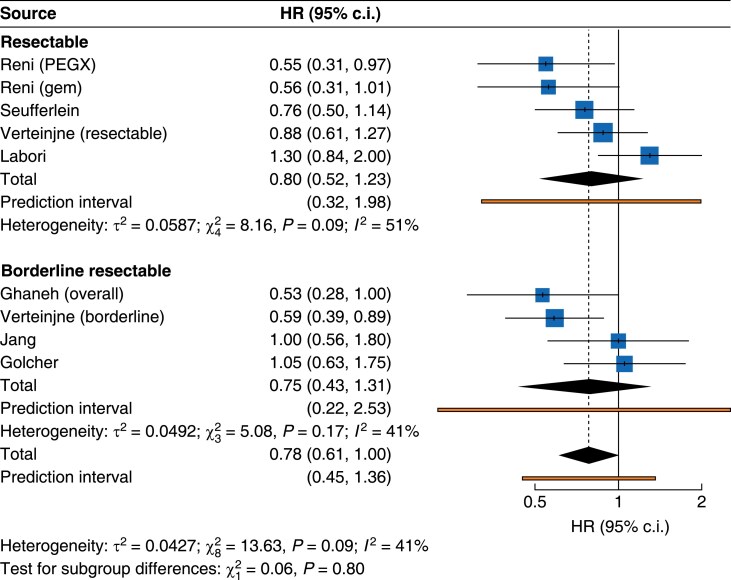
Forest plot demonstrating pooled analysis of overall survival with subgroup analysis according to resectability criteria (BR-PDAC *versus* R-PDAC) as per NCCN criteria PEXG, cisplatin, epirubicin, gemcitabine and capecitabine; BR-PDAC, borderline resectable pancreatic cancer; R-PDAC, resectable pancreatic cancer; NCCN, National Comprehensive Cancer Network.

**Fig. 4:**
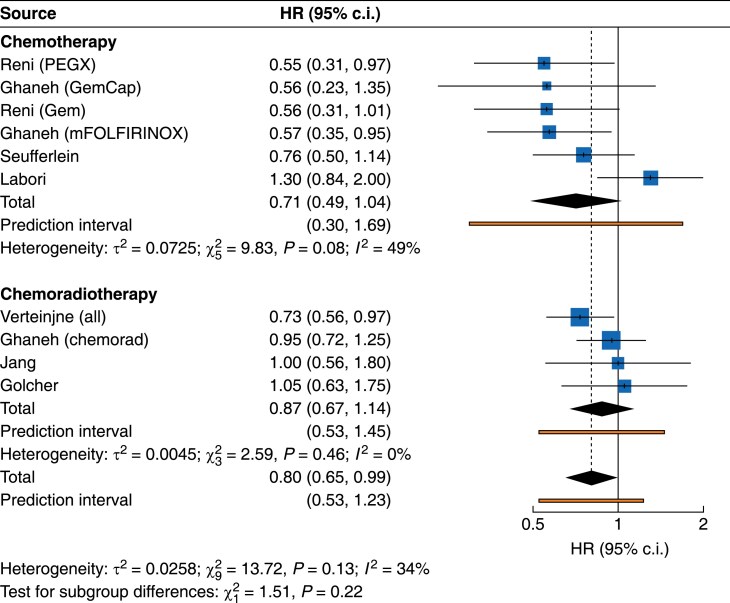
Forest plot demonstrating pooled analysis of overall survival with subgroup analysis according to treatment regimen (chemotherapy *versus* chemoradiotherapy) PEXG, cisplatin, epirubicin, gemcitabine and capecitabine; mFOLFIRINOX, folinic acid, flurouracil, irinotecan and oxaliplatin.

PFS was reported in six trials and was measured from the time of operation in ESPAC-5, point of randomization in PREOPANC, NEONAX and PACT-15, as well as by Golcher. In Jang, PFS was calculated from the start of first treatment. Neoadjuvant treatment resulted in longer PFS (HR 0.80, 95% c.i. 0.65 to 0.99; *P* = 0.041). Five trials reported median PFS (mPFS) with eight comparisons. mPFS ranged between 4.7 and 16.9 months. Five comparisons favoured neoadjuvant treatment and three upfront surgery. Neoadjuvant treatment did not improve PFS for the BR-PDAC (HR 0.75, 95% c.i. 0.43 to 1.31) or the R-PDAC (HR 0.80, 95% c.i. 0.52 to 1.23) cohorts (*Fig. 3* and *Table 3*). Influence assessment demonstrated NORPACT-1 was an outlier for heterogeneity and influence. Removal of this trial did not affect the pooled analysis for R-PDAC (HR 0.73, 95% c.i. 0.51 to 1.03). Neoadjuvant treatment did not improve PFS for chemotherapy (HR 0.71, 95% c.i. 0.49 to 1.04) or chemoradiotherapy (HR 0.87, 95% c.i. 0.67 to 1.14) subgroups (*Fig. 4* and *Table 3*). Influence assessment demonstrated NORPACT-1 was an outlier and exclusion resulted in a significant PFS advantage for those treated with neoadjuvant chemotherapy (HR 0.62, 95% c.i. 0.51 to 0.76) and a significant between subgroup difference (*P* = 0.002).

Resection rates were reported in all trials totalling 1194 patients. Resection rates were significantly lower overall in patients who received neoadjuvant therapy *versus* those who did not (72.6% *versus* 80.6%, RR 0.94, 95% c.i. 0.89 to 0.99; *P* = 0.02). This effect was driven by significantly lower resection rates in the chemoradiotherapy subgroup (59.6% *versus* 72.5%, RR 0.87, 95% c.i. 0.81 to 0.93; subgroup difference *P* = 0.001).

R0 rates were available for eight trials (nine analysis arms) with a higher rate in the neoadjuvant cohort relative to upfront surgery (43.8% *versus* 23.0%, RR 1.35, 95% c.i. 1.16 to 1.57; *P* = 0.002). Whilst all subgroups of patients receiving neoadjuvant therapy had higher rates of R0 resections than those receiving upfront surgery, the chemoradiotherapy subgroup was the only one to achieve statistical significance (43.2% *versus* 30.0%, RR 1.40, 95% c.i. 1.07 to 1.83). This was not significantly different from the chemotherapy cohort (*P* = 0.613).

N0 rates were available for eight trials (nine arms) and neoadjuvant treatment was associated with higher N0 rates (30.9% *versus* 15.0%, RR 2.03, 95% c.i. 1.50 to 2.74; *P* = 0.001). N0 rates in patients receiving neoadjuvant treatment were significantly higher in all subgroups except the R-PDAC cohort—chemoradiotherapy (RR 2.55, 95% c.i. 1.28 to 5.07), chemotherapy (RR 1.81, 95% c.i. 1.09 to 2.99) and BR-PDAC (RR 2.05, 95% c.i. 1.13 to 3.72). There were no significant differences between subgroups.

SAEs were reported fully in eight trials (nine arms) and were notably increased in the neoadjuvant treatment arms (56.1% *versus* 27.0%, RR 1.92, 95% c.i. 1.28 to 1.89; *P* = 0.007). SAEs were more common in the chemotherapy arm (63.5% *versus* 26.9%, RR 2.04, 95% c.i. 1.56 to 2.67) and in those with R-PDAC (57.0% *versus* 33.3%, RR 1.70, 95% c.i. 1.07 to 2.74).

Results for vascular resection rate, complications and rate of starting adjuvant therapy were non-significant and can be seen in *Table 4*.

**Table 4:** Table to summarize the secondary outcome analysis

Pooled characteristics		*N*	Patients	Upfront surgery	Neoadjuvant	Random-effects model			
				*n* = 592	*n* = 602				
				*n* (%)	*n* (%)	RR	95% c.i.	*I* ^2^	*P*
**Resection rate**	Overall	10	1194	477 (80.6)	437 (72.6)	0.94	0.89,0.99	0.0%	0.020
	CRT	5	432	159 (72.5)	127 (59.6)	0.82	0.79,0.86	0.0%	0.001
	CTx	5	763	319 (85.3)	310 (79.7)	0.96	0.91,1.02	0.0%	
	BR-PDAC	5	677	257 (78.7)	251 (71.5)	0.95	0.85,1.06	5.6%	0.377
	R-PDAC	5	517	220 (82.7)	186 (74.1)	0.91	0.85,0.97	0.0%	
**R0 rate**	Overall	9	832	136 (33.0)	184 (43.8)	1.35	1.16,1.57	0.0%	0.002
	CRT	5	432	66 (30.0)	92 (43.2)	1.40	1.07,1.83	0.0%	0.613
	CTx	4	401	71 (36.4)	92 (44.4)	1.31	0.95,1.80	0.0%	
	BR-PDAC	4	315	30 (20.5)	60 (35.5)	1.85	0.63,5.39	66.3%	0.243
	R-PDAC	5	517	106 (39.8)	124 (49.4)	1.22	0.91,1.65	14.4%	
**N0 rate**	Overall	9	1194	89 (15.0)	186 (30.9)	2.03	1.50,2.74	15.5%	0.001
	CRT	5	432	30 (13.7)	80 (37.6)	2.55	1.28,5.07	32.4%	0.223
	CTx	4	763	59 (15.8)	106 (27.2)	1.81	1.27,2.58	0.0%	
	BR-PDAC	4	564	40 (15.0)	93 (31.3)	2.05	1.13,3.72	8.2%	0.345
	R-PDAC	5	384	34 (17.2)	49 (26.3)	1.59	0.86,2.95	0.0%	
**Vascular resection rate**	Overall	6	430	44 (21.4)	37 (16.5)	0.67	0.47,0.97	0.0%	0.038
	CRT	3	148	17 (23.8)	16 (21.1)	0.88	0.64,1.20	0.0%	0.019
	CTx	3	283	27 (20.1)	21 (14.2)	0.55	0.24,1.23	0.0%	
	BR-PDAC	3	202	24 (27.6)	28 (24.3)	0.78	0.53,1.17	0.0%	0.011
	R-PDAC	2	228	20 (16.8)	9 (8.3)	0.44	0.03,6.29	0.0%	
**Complication rate**	Overall	9	600	105 (32.8)	101 (36.1)	1.04	0.72,1.52	41.5%	0.810
	CRT	5	286	76 (47.9)	73 (57.5)	1.21	0.97,1.85	23.4%	0.274
	CTx	4	315	29 (18.0)	28 (18.3)	0.79	0.25,2.49	45.9%	
	BR-PDAC	3	128	28 (45.2)	24 (36.4)	0.74	0.37,1.51	0.0%	0.433
	R-PDAC	4	308	31 (18.7)	28 (19.7)	1.04	0.29,3.70	46.7%	
**Significant adverse effects**	Overall	9	994	106 (27.0)	338 (56.1)	1.92	1.28,1.89	44.4%	0.007
	CRT	5	412	54 (27.2)	91 (42.7)	2.76	0.40,18.87	53.1%	0.622
	CTx	4	583	52 (26.9)	247 (63.5)	2.04	1.56,2.67	0.0%	
	BR-PDAC	4	497	24 (16.4)	195 (55.6)	3.14	0.51,19.25	58.3%	0.298
	R-PDAC	4	497	82 (33.3)	143 (57.0)	1.70	1.06,2.74	52.4%	
**Adjuvant therapy rate**	Overall	9	778	229 (55.6)	211 (57.7)	0.92	0.76,1.12	18.7%	0.353
	CRT	5	432	112 (51.0)	87 (40.8)	0.76	0.48,1.23	30.5%	0.165
	CTx	4	420	118 (60.7)	124 (59.9)	0.99	0.79,1.23	0.0%	
	BR-PDAC	3	202	40 (46.0)	47 (40.9)	0.86	0.66,1.11	0.0%	0.967
	R-PDAC	4	384	124 (62.6)	108 (58.1)	0.85	0.35,2.01	66.8%	

R-PDAC, resectable pancreatic cancer; BR-PDAC, borderline resectable pancreatic cancer; CRT, chemoradiotherapy; CTx, chemotherapy.

## Discussion

This review evaluated the role of neoadjuvant therapy in pancreatic cancer and updated the analysis of Van Dam *et al*.^43^ through inclusion of two further RCTs and the published results of ESPAC-5^44^. It complemented the comprehensive review by Springfield *et al.*^45^.

This meta-analysis demonstrated a significant survival benefit of neoadjuvant treatment over upfront surgery overall and in those with BR-PDAC. No improvement in survival was seen in trials recruiting patients with R-PDAC. This further supports National Institute of Clinical Excellence (NICE) guidance that neoadjuvant chemotherapy should be offered to those with BR-PDAC as part of a clinical trial and would suggest this should now be standard of care^46^. Improvement in PFS was only seen when pooling all studies, with no subgroup differences.

Both OS and PFS were influenced by the results of the NORPACT-1 trial, which contributed significantly to the influence and heterogeneity of the analysis. The reasons for NORPACT-1 being an outlier are likely multifactorial, but the use of unmodified FOLFIRINOX is an important difference affecting treatment rates. The per-protocol analysis of those with histopathological PDAC who received at least one cycle of neoadjuvant treatment did not demonstrate a difference in OS (HR 1.46, 95% c.i. 0.99 to 2.17; *P* = 0.058) or PFS (HR 1.22, 95% c.i. 0.73 to 2.04; *P* = 0.45). Modification of the FOLFIRINOX regimen is associated with a lower SAE rate and improved treatment completion rate as utilized in SWOG-1505 (87%) and PANACHE01-PRODIGE48 (89%) compared with 60% in NORPACT-1^35,47,48^. The difference in 18-month OS for the upfront surgery arm *versus* mFOLFIRINOX arm of NORPACT-1 (73% *versus* 60%) contrasts with those of FOLFIRINOX-based neoadjuvant trials for which survival was better for neoadjuvant therapy than upfront surgery at 12 months (FOLFIRINOX arm of ESPAC-5 (84% *versus* 39%) and PANACHE01-PRODIGE48 (84.1% *versus* 80.8%)). Given mFOLFIRINOX is standard of care the use of this in both neoadjuvant and adjuvant arms of PREOPANC-3 and Alliance A021806 will add further clarity to its benefit in the resectable setting^43,49^.

One of the principal concerns over neoadjuvant therapy is progression during its delivery; however, upfront surgery does not guarantee surgical resection. Neoadjuvant treatment should enable the selection of patients with biologically responsive tumours and prevent those who progress radiologically and/or biochemically from futile surgery. In the upfront surgery arm, 19.4% of patients receiving upfront surgery were not resected due to more advanced disease than radiologically suspected. Whilst resection rates were comparable in those with BR-PDAC and those treated with chemotherapy, the chemoradiotherapy cohort had significantly lower rates than those treated with chemotherapy (subgroup difference, *P* = 0.001), alluding to the inherent difficulty in staging and resecting tumours postneoadjuvant radiotherapy. The difficulties in radiological assessment have been highlighted in the PACT-UK study and their radiological proforma aims to improve the standardization of classification and improve decision-making at cancer multidisciplinary team meetings^50^.

In adjuvant trials, resection margin status and lymphatic involvement are correlated with OS^4,5^. The significantly increased N0 and R0 rates associated with neoadjuvant treatment in this meta-analysis may be indicative of disease downstaging and potentially drivers of improved long-term survival. This hypothesis is supported by the outcomes of PREOPANC-1 in which mOS was only improved by 1.4 months but in the long-term analysis 14% more of the patients were alive at 5 years with neoadjuvant chemoradiotherapy (20.5% *versus* 6.5%)^39^. In this trial N0 and R0 rates were significantly lower in the neoadjuvant cohort (both *P* < 0.001). The impact of these variables on long-term outcomes in NORPACT-1, ESPAC-5 and PANACHE01-PRODIGE48 cohorts will be of great interest^35,37,48^.

As demonstrated in the differences between FOLFIRINOX and mFOLFIRINOX, tolerability of treatment is vital to balance against any improvements in survival. SAEs were noted more frequently in the neoadjuvant treatment cohort, particularly the chemotherapy cohort. As expected, FOLFIRINOX/mFOLFIRONOX appeared to have more treatment-related side effects than gemcitabine-based neoadjuvant treatment^35^.

The use of chemoradiotherapy in pancreatic cancer remains controversial and this subgroup analysis did not demonstrate an OS or PFS benefit. It also showed a significantly worse resection rate compared to trials of chemotherapy (*P* = 0.001). Although it did not have a comparator arm to direct surgery, Alliance A021501 randomized patients to neoadjuvant mFOLFIRINOX with or without hypofractionated radiotherapy (33–40 Gy), resulting in an improved mOS in the chemotherapy-only cohort (29.8 *versus* 17.1 months)^51^. This finding supported that of ESPAC-5 which demonstrated a significant improvement in survival of the chemotherapy cohort *versus* surgery (HR 0.22, 95% c.i. 0.09 to 0.52; *P* = <0.001) that was not seen in chemoradiotherapy *versus* surgery (HR 0.45, 95% c.i. 0.17 to 1.20; *P* = 0.100).

Despite completion of adjuvant treatment being the strongest predictor of long-term survival, adjuvant treatment rates on average in the UK are 62%^18^. In this analysis there was no difference in adjuvant treatment rates between those receiving neoadjuvant therapy and upfront surgery (57.7 *versus* 55.6%; *P* = 0.353). To counter the drop-off in treatment after surgery, total neoadjuvant treatment has been tested in PREOPANC-2, which compared total neoadjuvant treatment with FOLFIRINOX *versus* gemcitabine-based chemoradiotherapy in both BR-PDAC and R-PDAC^52^. According to the European Society for Medical Oncology (ESMO) abstract presentation, resection rates were comparable between arms (77% *versus* 75%) and comparable with the neoadjuvant arm of this pooled analysis (72.6%). There was no difference in mOS after 41.7 months follow-up (21.9 months *versus* 21.3 months, HR 0.87, 95% c.i. 0.68 to 1.12; *P* = 0.28) and treatment SAEs were comparable (49% *versus* 43%; *P* = 0.23). In context with the chemoradiotherapy arm of PREOPANC-1 (mOS 15.7 months) this full article will be insightful in guiding further trial design.

This meta-analysis was performed on a large number of patients recruited to high-quality trials with low bias and utilized intention-to-treat analysis. Despite this it is limited by the heterogeneity within treatment regimens of the trials, especially as the evidence base has changed with time. Differing definitions and start times for PFS limit effective characterization and would benefit from standardization in future research. The definition of surgical resectability has also altered over time and is not universally agreed upon, with two trials needing reclassifying. Whilst able to be included from abstract form, the full article of PREP-2/JSAP-05 needs publication to allow for more critical review. Further, there are other ongoing trials investigating neoadjuvant treatment comparative to upfront surgery and these studies could not be included in this review as they have not yet been published with results in a format that would enable pooled analysis (JASPAC and PANACHE01-PRODIGE48)^38,48^.

This meta-analysis demonstrated a survival benefit of neoadjuvant treatment especially for BR-PDAC. Neoadjuvant treatment should be considered standard of care for patients with BR-PDAC disease. Further trials are needed to identify the optimal regimens before and after surgery. Standardized definitions of resectability are vital in future RCTs.

## Supplementary Material

zrae172_Supplementary_Data

## Data Availability

On request.
