# Supplementary material for: Neoadjuvant treatment versus upfront surgery in borderline resectable and resectable pancreatic ductal adenocarcinoma: meta-analysis
Source: BJS Open. 2025 Mar 24;9(2):zrae172. doi: 10.1093/bjsopen/zrae172 (PMC11932015; doi:10.1093/bjsopen/zrae172)
Supplement: zrae172_Supplementary_Data [file zrae172_supplementary_data.zip › Supplementary_Material (2).docx]

**A meta-analysis of neoadjuvant treatment versus upfront surgery in borderline resectable and resectable pancreatic ductal adenocarcinoma**

*Luke D Dickerson ^1,2^, Jayden Gittens ^1^, Chris Brunning ^1^, Richard Jackson ^1^, Michael C Schmid ^1^, Ainhoa Mielgo ^1^, Daniel Palmer ^1,3^, Christopher M Halloran ^1,2^, Paula Ghaneh ^1,2^*

*1: Institute of Systems, Molecular and Integrative Biology, University of Liverpool, UK,*

*2: Pancreatobiliary Surgery Unit, Liverpool University Foundation Trust, UK,*

*3: Clatterbridge Cancer Centre, Liverpool, UK*

**Corresponding author.** ***:*** *Mr Luke Dickerson , Pancreatobiliary Surgery Unit, Royal Liverpool University Hospital, Liverpool, L7 8XP, [luke.dickerson@liverpool.ac.uk](mailto:luke.dickerson@liverpool.ac.uk)*

**ORCID ID**; [**https://orcid.org/0000-0001-6281-8658**](https://orcid.org/0000-0001-6281-8658)

**Twitter** @MrLDDickerson

**Supplementary Materials - Index**

| **Supplementary Tables** |  |
| --- | --- |
| Table S1: Anatomical Classifications of Resectability | *page 2* |
| Table S2: Search terms, inclusion and exclusion criteria | *page 3* |
| **Supplementary Figure** |  |
| Fig S1: Prisma diagram | *page 4* |
| Fig S2A: Baujat Plot  FigS2B: Influence Assessment  Fig S2C: Leave-one-out analysis (Effect size)  Fig S2D: Leave-one-out analysis (I2) | *page 4*  *page 5*  *Page 6*  *Page 7* |

**Supplementary Tables**

| **Classification** | **AHPBA/SSAT/SSO** ^7^ | **MD Anderson** ^8,9^ | **Alliance** ^10^ | **NCCN ^6^** |
| --- | --- | --- | --- | --- |
| **Superior Mesenteric / Portal Vein** | | | | |
| **Resectable** | No abutment, encasement or occlusion | Abutment, encasement without occlusion | Interface between tumour and vessel <180ᵒ | No tumour contact or ≤180° contact without vein contour irregularity |
| **Borderline Resectable** | Abutment, encasement or occlusion | Occlusion | Interface between tumour and vessel ≥180ᵒ and/or reconstructable occlusion | Solid tumour contact measuring >180°, or solid tumour contact ≤180° with contour irregularity or thrombosis |
| **Unresectable / Locally Advanced** | Unreconstructable | Unreconstructable | Unreconstructable | Unreconstructable |
| **Superior Mesenteric Artery** | | | | |
| **Resectable** | No abutment | No abutment | No interface between tumour and vessel | No solid tumour contact |
| **Borderline Resectable** | Abutment | Abutment | Interface between tumour and vessel <180ᵒ | Solid tumour contact ≤180° |
| **Unresectable / Locally Advanced** | Encasement | Encasement | Interface between tumour and vessel ≥180ᵒ | Solid tumour contact >180° |
| **Common Hepatic Artery** | | | | |
| **Resectable** | No abutment or encasement | No abutment or encasement | No interface between tumour and vessel | No solid tumour contact |
| **Borderline Resectable** | Abutment or short-segment encasement | Abutment or short-segment encasement | Reconstructable, short-segment interface between tumour and vessel (of any degree) | Solid tumour contact without extension to the coeliac artery or hepatic artery bifurcation |
| **Unresectable / Locally Advanced** | Unreconstructable | Unreconstructable | Unreconstructable | Unreconstructable |
| **Coeliac Axis** | | | | |
| **Resectable** | No abutment or encasement | No abutment or encasement | No interface between tumour and vessel | No solid tumour contact |
| **Borderline Resectable** | No abutment or encasement | Abutment | Interface between tumour and vessel <180ᵒ | Solid tumour contact ≤180° |
| **Unresectable / Locally Advanced** | Abutment or encasement | Encasement | Interface between tumour and vessel ≥180ᵒ | Solid tumour contact >180° |
| **Supplementary Table S1:** Table to demonstrate differences in anatomical classifications of resectable, borderline resectable and unresectable / locally advanced pancreatic cancer | | | | |

| **Database** | **Search Date** | **Search Term** | **Number of Records** |
| --- | --- | --- | --- |
| Pubmed | 01/05/2024 | (((((Pancreatic OR Pancreas) AND (Cancer OR carcinoma or adenocarcinoma or neoplasm)) AND (neoadjuvant or pre-operative)) AND (chemotherapy or radiotherapy or chemoradiotherapy)) AND (surgery or operative or operation)) AND (randomised OR allocated OR randomized) | 310 |
| SCOPUS | 01/05/2024 | ( TITLE-ABS-KEY ( pancreatic OR pancreas ) AND TITLE-ABS-KEY ( carcinoma OR adenocarcinoma OR cancer OR neoplasm ) AND TITLE-ABS-KEY ( neoadjuvant OR pre-operative ) AND TITLE-ABS-KEY ( chemotherapy OR chemoradiotherapy OR radiotherapy ) AND TITLE-ABS-KEY ( surgery OR operative OR operative ) AND TITLE-ABS-KEY ( randomised OR randomized OR allocated ) OR TITLE-ABS-KEY ( controlled AND clinical AND trial OR rct OR cross-over OR double AND blind ) ) | 385 |
| Cochrane Central Register of Controlled Trials | 01/05/2024 | (pancreas OR pancreatic) AND (cancer OR carcinoma OR neoplasm OR adenocarcinoma) AND (neoadjuvant OR preoperative) AND (Surgery OR operation OR operative) AND (Chemotherapy OR Chemoradiotherapy OR radiotherapy) | 440 |
| **Total** | | | **1135** |
| **Total After Deduplication** | | | **880** |
| **Inclusion:** | RCT only | | |
|  | Direct comparison of treatment to surgery | | |
|  | Pancreas Cancer only [pancreatic adenocarcinoma & pancreatic ductal adenocarcinoma & ampullary adenocarcinoma] | | |
| **Exclusion:** | Other types of pancreatic cancer (cholangiocarcinoma etc) | | |
|  | No direct comparison to surgery | | |
|  | Patients with metastatic disease | | |
|  | Trials that are not randomised | | |
| **Supplementary Table S2**: Table with specific search terms utilised for each database and inclusion and exclusion criteria | | | |

**Supplementary Figures:**

**
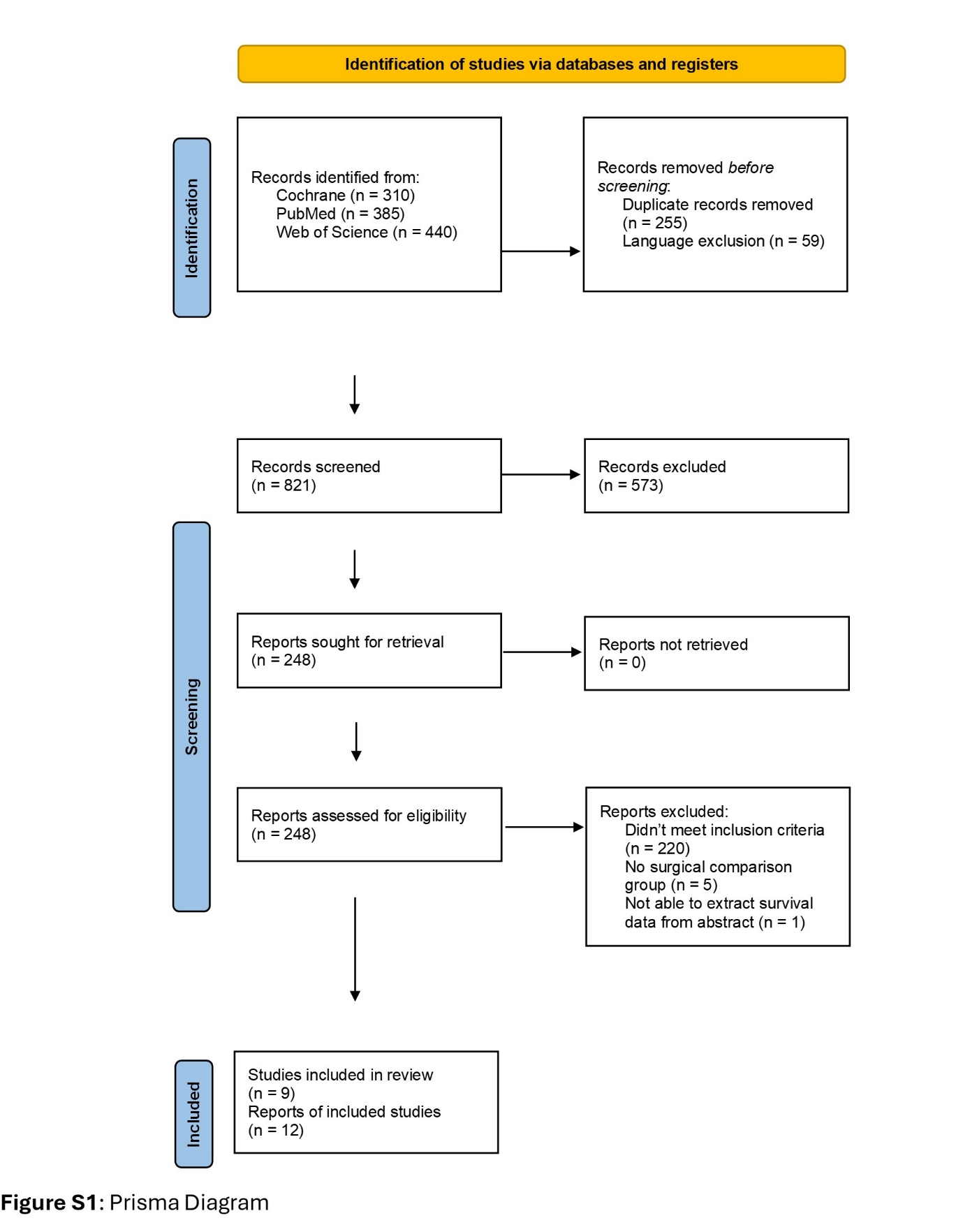
**

**Supplementary Figure 2A:** Baujat chart to demonstrate trial characteristics by influence on result and contribution to heterogeneity

**
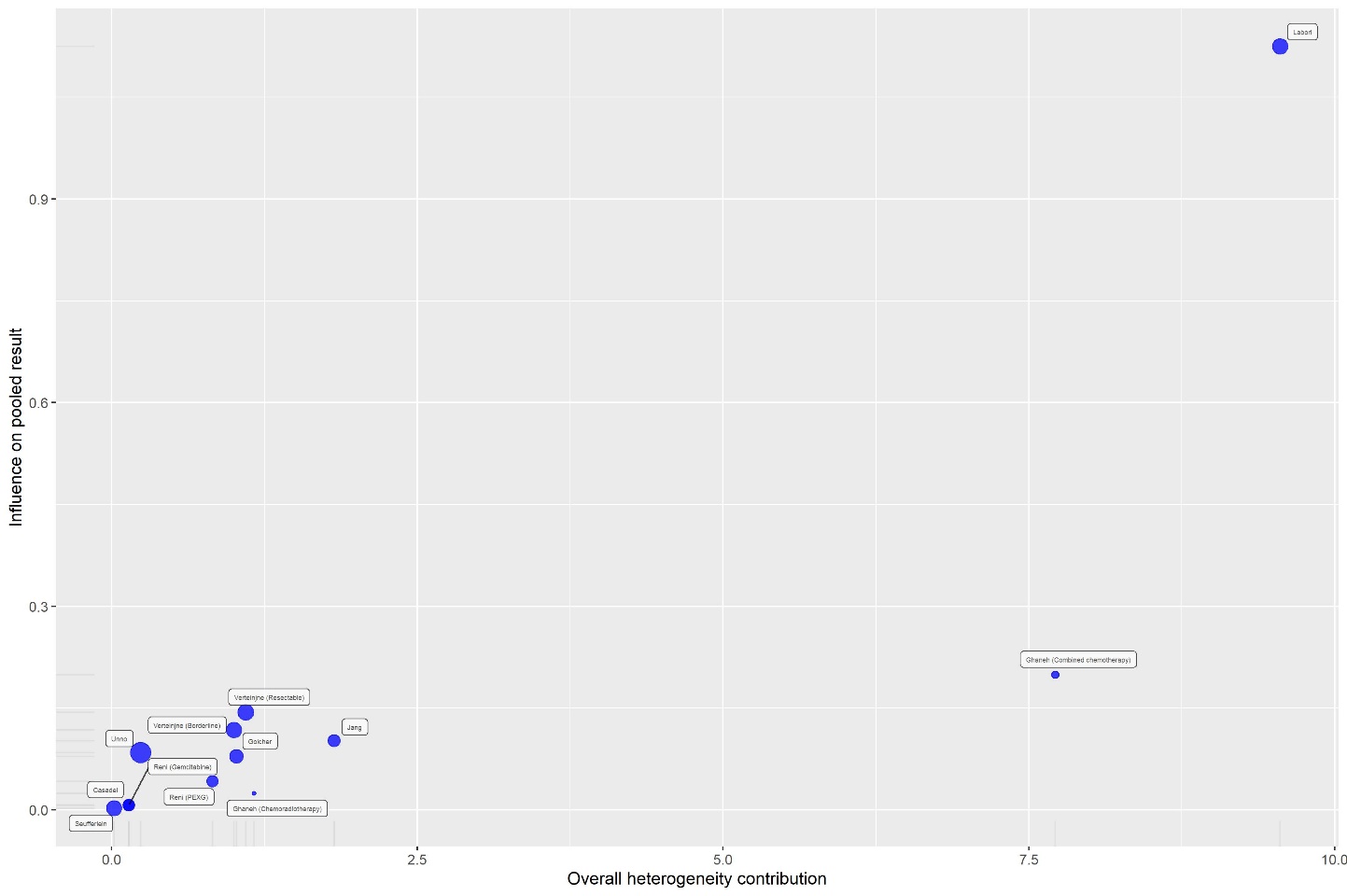
**

**Supplementary Figure 2B**: Influence assessment for trials to demonstrate their effect on outcome and heterogeneity

**
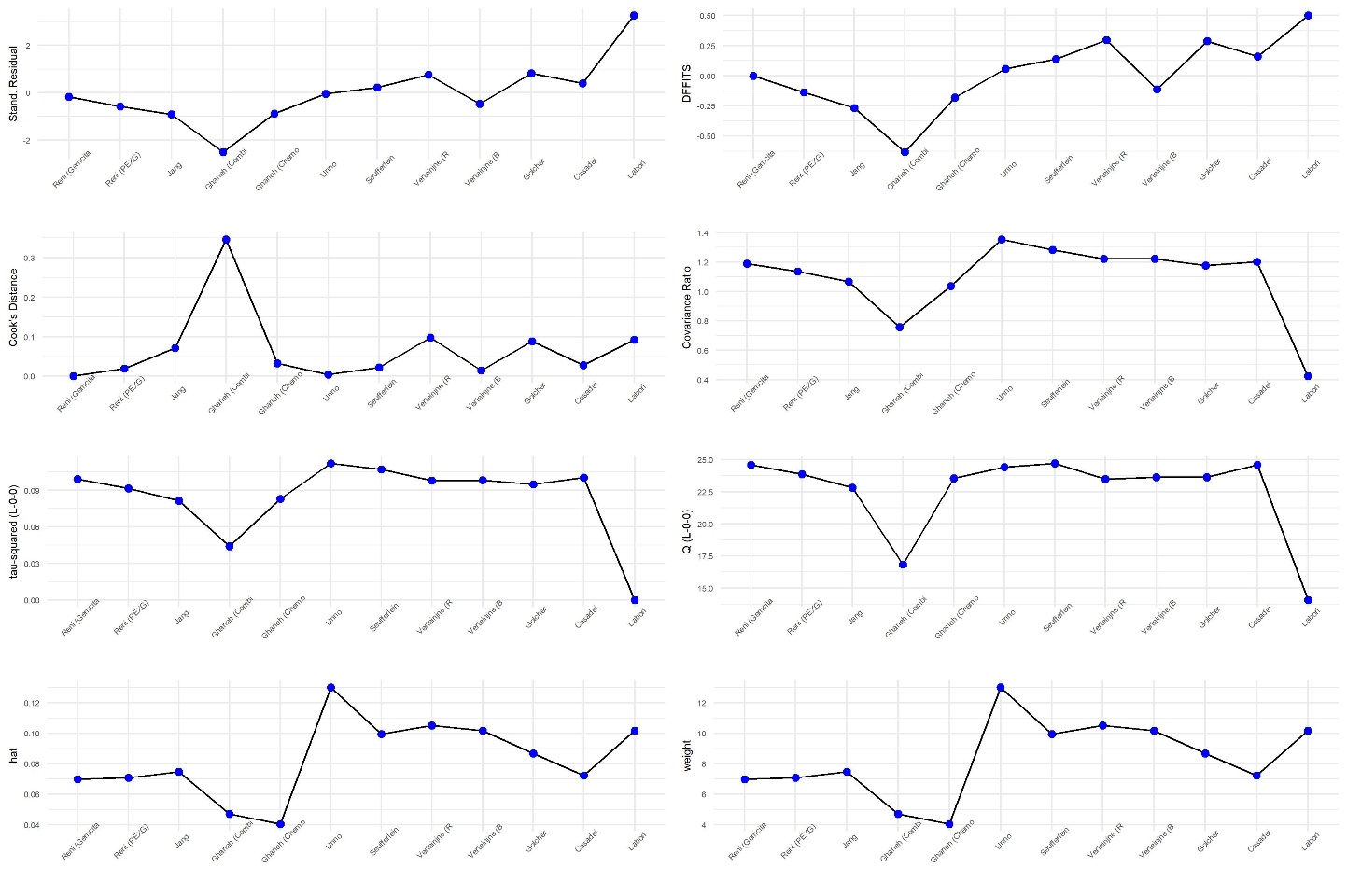
**

**Supplementary Figure 2C**: Forest plot to demonstrate the leave-one-out analysis sorted by effect size.

**
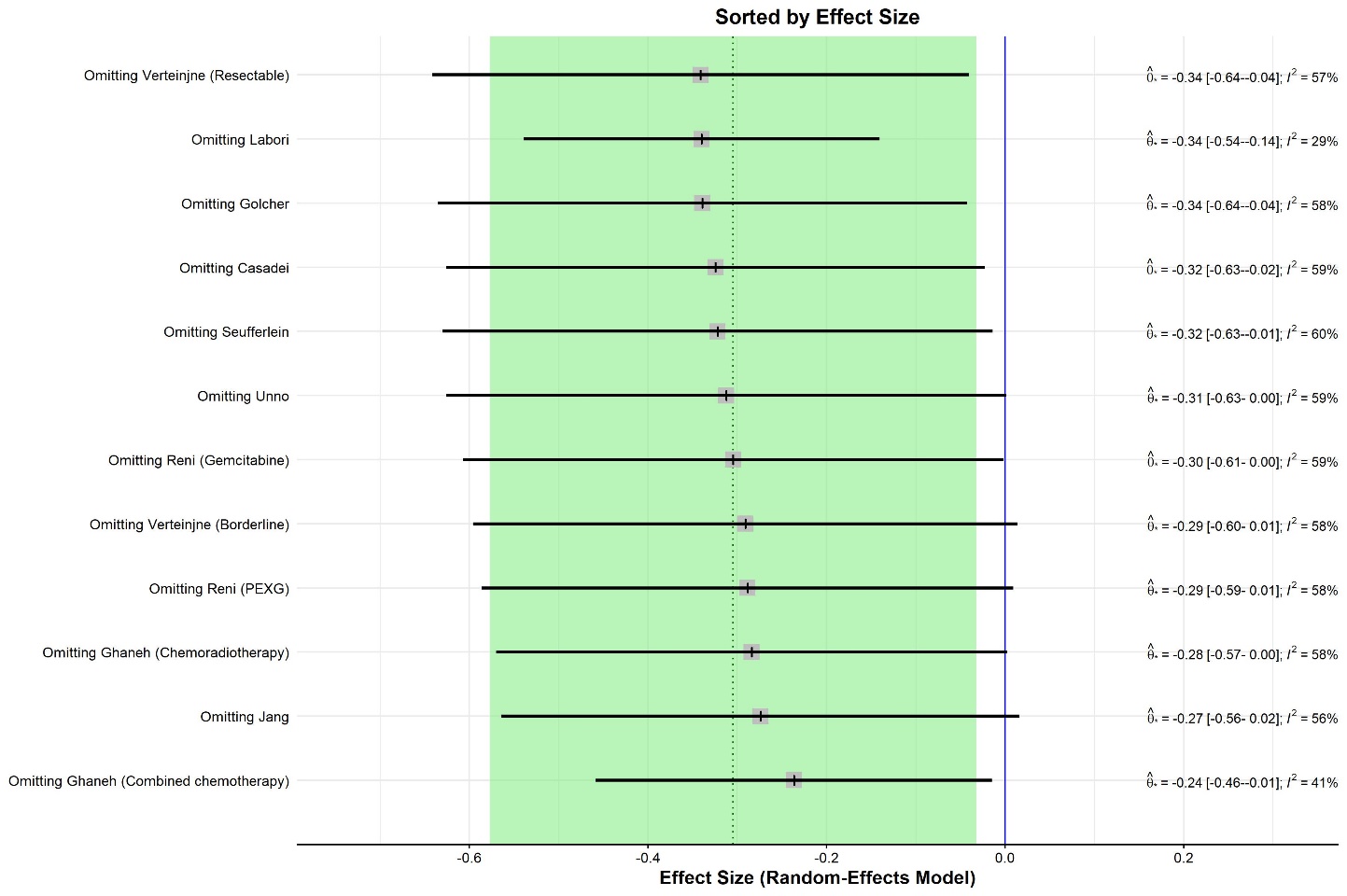
**

**Supplementary Figure 2D:** Forest plot to demonstrate the leave-one-out analysis sorted by effect on heterogeneity as measured by I2.

**
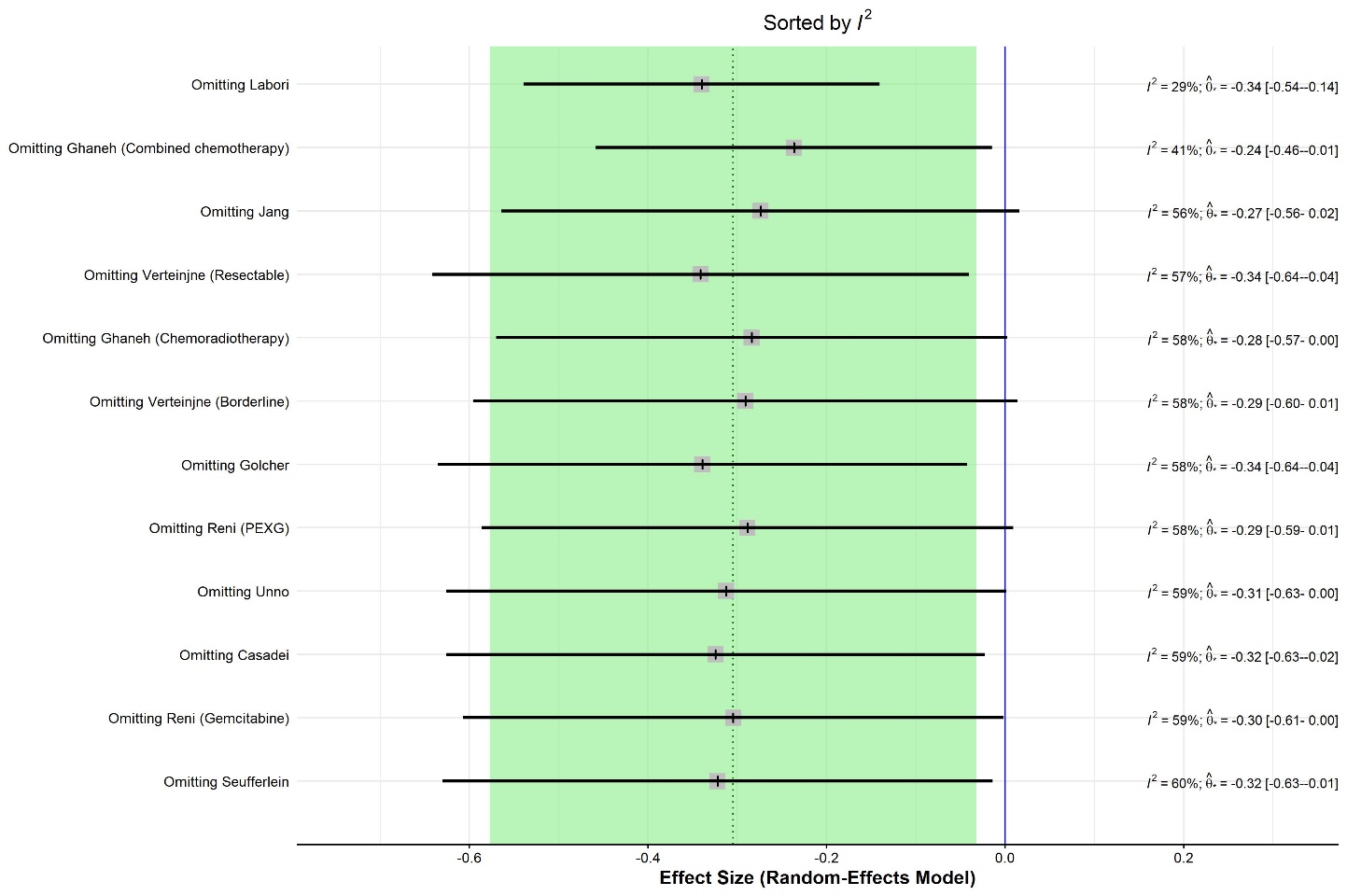
**
