# Supplementary figures and images for: Neoadjuvant treatment versus upfront surgery in borderline resectable and resectable pancreatic ductal adenocarcinoma: meta-analysis
Source: BJS Open. 2025 Mar 24;9(2):zrae172. doi: 10.1093/bjsopen/zrae172 (PMC11932015; doi:10.1093/bjsopen/zrae172)

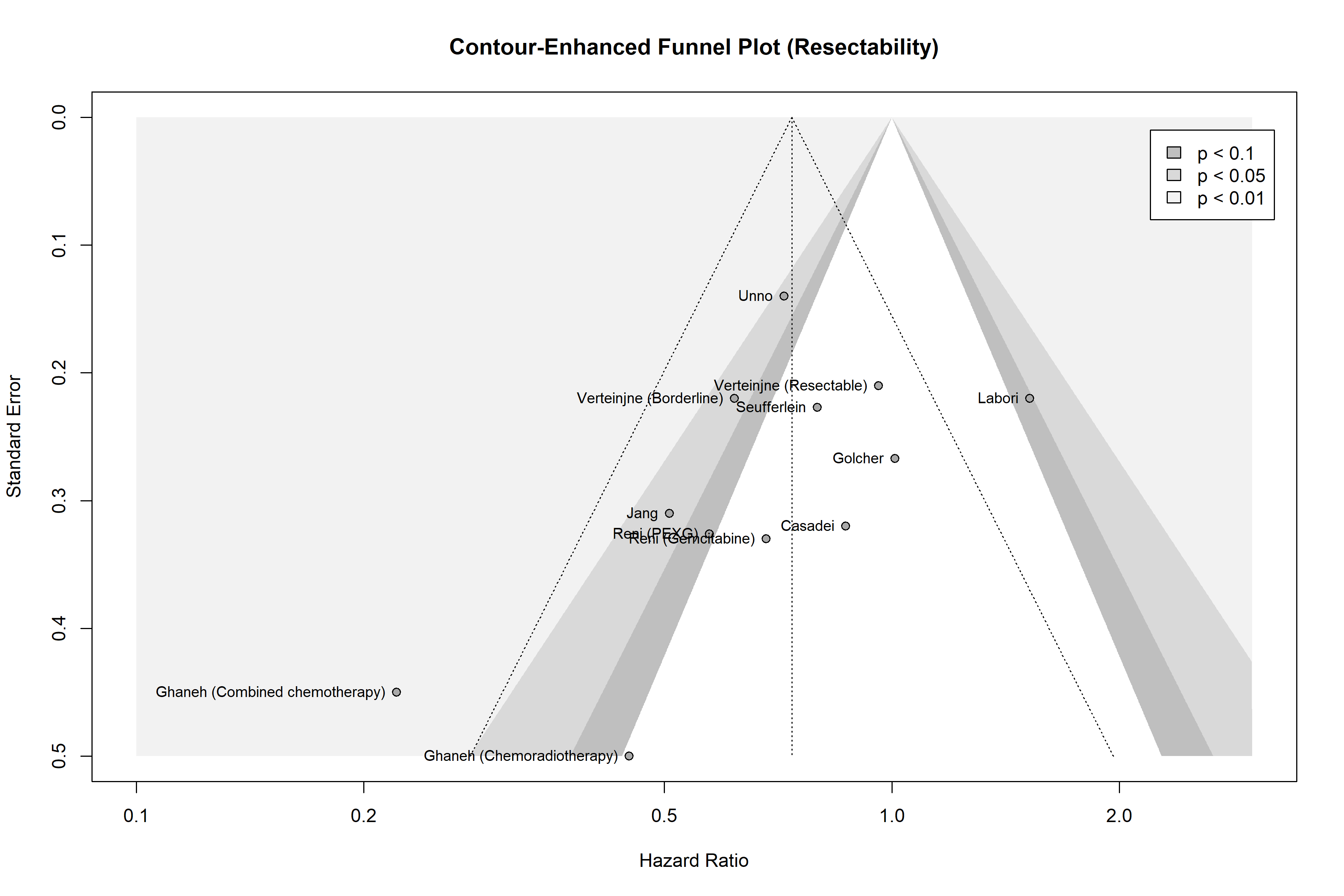

Supplement: zrae172_Supplementary_Data [file zrae172_supplementary_data.zip › PublicationBias.jpg]
